# A moderate dose of alcohol selectively reduces empathic accuracy

**DOI:** 10.1007/s00213-018-4859-y

**Published:** 2018-02-28

**Authors:** Freya Thiel, Brian D. Ostafin, Jana R. Uppendahl, Lenka J. Wichmann, Marco Schlosser, Marije aan het Rot

**Affiliations:** 1 Department of Psychology, University of Groningen, Grote Kruisstraat 2/1, 9712 TS Groningen, Netherlands; 2 Department of Clinical, Neuro-, and Developmental Psychology, VU University Amsterdam, Van der Boechorststraat 1, 1081 BT Amsterdam, The Netherlands; 3 Division of Psychiatry, University College London, 149 Tottenham Court Road, London, W1T 7NF UK; 4 School of Behavioral and Cognitive Neurosciences, University of Groningen, Groningen, Netherlands

**Keywords:** Alcohol consumption, Empathic accuracy, Risk for alcoholism, Social cognition

## Abstract

**Rationale:**

Drinking alcohol is associated with various interpersonal effects, including effects on cognitive empathy. Empathic accuracy (EA) is a form of cognitive empathy concerned with perceivers’ accuracy in inferring a target’s thoughts and feelings. The effects of alcohol on EA have not previously been studied.

**Objectives:**

We examined the effect of a moderate alcohol dose on EA in social drinkers.

**Methods:**

Fifty-four men with varying levels of hazardous drinking according to the Alcohol Use Disorders Identification Test (AUDIT) participated in a randomized, double-blind, between-group study. The alcohol group received 0.56 g/kg alcohol in a vodka and tonic-mixed drink. The placebo group received tonic, with 4 ml of vodka sprayed on top. All participants performed an EA task that involved watching 16 videos of people narrating positive and negative emotional autobiographical events and continuously rating how targets felt while narrating.

**Results:**

There were no significant main effects of beverage condition on the EA task. There was an effect of the condition by AUDIT interaction for EA on the positive videos. Post-hoc simple contrasts revealed that in participants with lower AUDIT scores, the alcohol condition had lower EA for positive videos than the placebo condition. No significant main effect for condition occurred in the participants with higher AUDIT scores.

**Conclusions:**

The effect of condition in participants with lower AUDIT scores indicates alcohol selectively reduced EA in individuals low on hazardous drinking. This suggests either alcohol-induced impairments of EA for positive events or a positivity bias in men at low risk for alcohol dependency.

**Electronic supplementary material:**

The online version of this article (10.1007/s00213-018-4859-y) contains supplementary material, which is available to authorized users.

## Introduction

Alcohol consumption can have both positive and negative effects. Higher alcohol doses have been widely associated with aggression (e.g., Bushman and Cooper 1990; Hoaken and Stewart 2003; Giancola and Parrott 2008; Pihl and Sutton 2009; Attwood and Munafo 2014). In contrast, low to moderate doses may contribute to positive mood and disinhibition, and alter the processing of social stimuli (e.g., Monahan and Lannutti 2000; additional references below). In line with these findings, in the present study we examined the effect of a moderate alcohol dose on social cognition using an empathic accuracy (EA) task.

The acute or immediate effects of alcohol on social cognition have often been studied using facial emotion recognition (FER) tasks, during which research participants recognize the emotions displayed in facial expressions presented in static images (Attwood et al. 2009; Craig et al. 2009; Attwood and Munafo 2014). However, as social cognition usually involves dynamic information processing, EA tasks may provide greater ecological validity. EA can be assessed by asking perceivers, i.e., participants, to watch previously recorded video clips of people, i.e., targets, narrating positive and negative emotional autobiographical events. Perceivers continuously rate how the target felt while discussing the event. The correlation between perceivers’ ratings and targets’ own ratings is then defined as a measure of EA (Hogenelst et al. 2015a). EA thus comprises one’s ability to accurately deduce and judge another’s thoughts and emotions based on verbal and nonverbal behavior, is recognized as a form of cognitive empathy (Ickes et al. 1990; Ickes 1993), and closely related to Theory of Mind (ToM) and perspective taking (Shamay-Tsoory et al. 2009). Daily social interactions comprise dynamic facial expressions and auditory information, with the latter having been found to be at least as important as visual information for the ability to recognize how another person is feeling (Zaki et al. 2009; Zaki and Ochsner 2012). By utilizing more naturalistic stimuli than FER tasks do, data obtained from EA tasks offers higher ecological validity (Zaki and Ochsner 2009; Hogenelst et al. 2015a, b). Moreover, EA has previously been found to be sensitive to intranasal oxytocin administration (Bartz et al. 2010), a pharmacological intervention aiming to increase brain levels of oxytocin, a hormone thought to regulate social behavior. The EA task used in this study was developed by Zaki et al. (2008). This supports our use of this EA task in the current alcohol administration study. A Dutch-language version was previously developed by aan het Rot and Hogenelst (2014).

An extensive body of previous research also suggests chronic alcohol misuse to be associated with impairments in social cognition. For instance, those with alcohol use disorder (AUD) have been reported to perform worse on FER tasks when compared to healthy controls (D’Hondt et al. 2014; Donadon and Osório 2014; Uekermann and Daum 2008). Two recent meta-analyses focused on the effects of chronic alcohol misuse on social cognition, both indicating large overall FER deficits in individuals with AUD compared to healthy controls (Castellano et al. 2015; Bora and Zorlu 2016). Bora and Zorlu further report specific deficits for recognition of anger and disgust in AUD, as well as impairments in decoding and reasoning aspects of ToM. While ToM decoding refers to the ability to infer another’s state of mind from perceptual information (e.g., facial expressions), ToM reasoning refers to the ability to infer another’s beliefs and intentions (Bora and Zorlu 2016). Overall, previous findings suggest evidence for overall social cognition deficits related to chronic alcohol misuse, as well as the possibility of specific impairments for recognition of negative emotions.

To date, only few prior studies have investigated the effects of alcohol consumption on social cognition. Alcohol activates neural reward systems related to positive affect, is associated with disinhibition and subjective relaxation, and can alter social stimuli processing (Fromme and D’Amico 1999; Dolder et al. 2017). Findings from FER studies indicate that in social drinkers, low to moderate alcohol doses (0.2 to 0.4 g/kg) lead to impairments in the recognition of sad but not angry or happy facial expressions (Attwood et al. 2009; Craig et al. 2009; Attwood and Munafo 2014). Kano et al. (2003) reported the discrimination of happy faces to be better after administration of a low dose (0.14 g/kg) compared to a moderate dose (0.56 g/kg) of alcohol. A recent study reported alcohol doses of 0.24 g/kg for women and 0.29 g/kg for men to facilitate happy face recognition (Dolder et al. 2017). Taken together, findings from these studies suggest that alcohol doses between 0.14 and 0.29 g/kg can enhance the processing of positive emotional stimuli, while 0.4 and 0.56 g/kg alcohol may impair processing of both positive and negative emotional stimuli. Nevertheless, other experimental studies reported no such effects of alcohol on emotion recognition at doses between 0.17 and 0.8 g/kg (Kamboj et al. 2013; Felisberti and Terry 2015). This current inconsistency of findings concerning the acute effects of alcohol consumption on social cognition may be explained by (1) differing alcohol doses and (2) the reliance on FER tasks as these tasks involve static and fairly artificial stimuli. We therefore investigated the effects of a moderate dose of alcohol on social cognition using an EA task.

### The present study

The present placebo-controlled study examined the effects of 0.56 g/kg alcohol on EA and the potential moderating effects of hazardous drinking. The same (Dutch language) EA task was previously used in an acute tryptophan depletion study (Hogenelst et al. 2015b) and designed after the (English language) EA task previously used in the intranasal oxytocin administration described previously (Bartz et al. 2010). As previous studies have shown higher EA for positive video clips (e.g., Zaki et al. 2008; Hogenelst et al. 2015b), we analyzed positive and negative videos separately. Based on previous findings from FER studies (e.g., Kano et al. 2003; Craig et al. 2009; Kamboj et al. 2013; Felisberti and Terry 2015; Dolder et al. 2017), we expected an alcohol dose of 0.56 g/kg to be associated with lower EA for both positive and negative emotional target videos, compared to a placebo group.

Given the association between chronic alcohol misuse and impairments in social cognition and empathy, we investigated hazardous drinking as a moderator. We hypothesized two possible outcomes for the effect of condition (alcohol versus placebo) on EA based on the low level of response model (LLRM), which posits that people whose reactions to alcohol are limited have a higher risk for developing alcoholism, and the differentiator model (DM), which posits that people who exhibit acute sensitization to alcohol are at higher risk for alcoholism (see Morean and Corbin 2010 for a review). Thus, on the one hand and in line with the LLRM, alcohol was expected to significantly decrease EA in participants with a low risk for alcohol dependence (i.e., non-hazardous drinkers). Participants with a high risk (i.e., hazardous drinkers) would not be affected, due to their reduced sensitivity to alcohol effects. On the other hand, conforming to the DM, alcohol might selectively decrease EA in hazardous drinkers.

## Methods

### Ethics statement

The Ethics Board of the Department of Psychology at the University of Groningen approved the study. Following a verbal and written study explanation, participants provided written informed consent. The study was conducted in accordance with the Declaration of Helsinki.

### Participants

Participants were 54 men ranging in age between 19 and 57 years (*M* = 24.6, *SD* = 6.49). They were recruited via posters in university buildings and local supermarkets, as well as online via local Facebook groups. Participants were pre-screened using an online questionnaire (Qualtrics, Provo, UT). Inclusion criteria based on self-report included (a) no evidence of problematic alcohol use (i.e., current or past treatment for alcohol use disorders or past diagnosis of alcohol dependence), (b) no current signs of alcohol dependence as indicated by a score of 2 or lower on the Short Michigan Alcohol Screening Test (SMAST), (c) no current use of contraindicated prescription medications (e.g., monoamine oxidase inhibitors), (d) no contraindicated medical conditions (e.g., diabetes, liver disease, pancreatitis, ulcer), and (e) no known neurological or physical impairments that may affect psychomotor abilities.

We subsequently contacted those meeting the inclusion criteria via email to arrange an appointment for a test session. Out of the 78 men, 54 responded and participated in the study (see Supplementary Material for a flow diagram). They were asked to refrain from drinking alcohol in the 24 h prior to the appointment and from eating a large meal in the preceding 2 h. A test session lasted around 2.5 h for which participants were reimbursed 20 euros.

### Questionnaires

To assess participants for alcohol use disorders, we administered the Dutch version of the Alcohol Use Disorders Identification Test (AUDIT; Saunders et al. 1993; Schippers and Broekman 2010). The AUDIT consists of 10 self-report items with either three or five answer choices, all of which are scored from 0 to 4, resulting in possible overall scores ranging between 0 and 40. A cutoff score of 8 has been shown to result in sufficient sensitivity and specificity in screening for a “strong likelihood of hazardous or harmful alcohol consumption” in general, as well as in each of the subscales *Hazardous Alcohol Use*, *Dependence Symptoms*, and *Harmful Alcohol Use*. The AUDIT was developed using a wide cross-national sample and has been shown to have high validity and reliability in different cultural contexts (Allen et al. 1997). We excluded items 9 and 10 from the current analyses due to having provided incorrect answer options. The internal consistency of the revised AUDIT (r-AUDIT; total score of items 1–8, excluding items 9 and 10) was acceptable, as indicated by a Cronbach coefficient α of 0.75.

To assess habitual drinking behavior and number of daily drinks, we used a Quantity-Frequency measure, asking participants to indicate the mean and peak number of drinks they consumed on each of the 7-week days during the last month (US National Institute on Alcohol Abuse and Alcoholism [NIAAA] 2003). Further, we administered the Drinking Motives Questionnaire (DMQ-R; Cooper 1994) to assess participants’ motives for drinking alcohol. The DMQ-R’s items load onto four factors that describe the main motives for drinking, namely *social*, *coping*, *enhancement*, and *conformity*. Furthermore, we included a single question to assess participants’ urge to drink alcohol. Participants read the statement “At the moment, I would like to drink alcohol [e.g. beer, wine, cocktail etc.].” and rated it on a 10-point Likert scale ranging from “not at all” (0) to “very much” (10). Participants answered this question four times, namely after entering the lab, immediately prior to beverage consumption, immediately after beverage consumption and prior to the EA task, and upon completion of the EA task.

For descriptive reasons, we included the Social Interaction Anxiety Scale (SIAS) to assess fear of social interaction. The SIAS is a self-report questionnaire, containing 19 items each rated on a 5-point Likert scale. High levels of internal consistency and test-retest reliability have been reported for the SIAS (Mattick and Clarke 1998).

### Empathic accuracy task

To assess emphatic accuracy (EA), we used a computer task developed by aan het Rot and Hogenelst (2014) after a similar task previously employed by Zaki et al. (2008). The task includes two previously validated sets of 20 video clips in which male and female targets recount positive or negative personal experiences (aan het Rot and Hogenelst 2014). During the EA task, participants (perceivers) watched 16 randomly chosen video clips of one of the two sets, such that the task lasted around 30 min in total; around 2 min per clip. The clips were presented in pseudo-random order, not showing the same target more than twice, or more than two positive or negative videos in a row.

Perceivers continuously rated how targets felt by using a dial, which corresponded to a 9-point Likert scale, anchored from 1 (extremely negative), over 5 (neutral), to 9 (extremely positive). Targets’ ratings of their own video clips had previously been gathered by aan het Rot and Hogenelst (2014).

We averaged continuous rating data of participants and targets across 5-s intervals. The first and final 5 s of all ratings were discarded. We performed data transformation in SAS 9.3 for Windows (SAS, Cary, NC) using the Yule-Walker method (see aan het Rot and Hogenelst 2014). For each video clip, we correlated perceiver ratings with target ratings and transformed correlation coefficients. These correlation coefficients *r* underwent Fisher *z* transformation prior to data analysis. We used these Fisher *z* scores for hypothesis testing.

### Alcohol dose and beverage administration

Participants in the alcohol condition (*n* = 28) received a moderate dose of 0.56 g of alcohol per kg body weight. This was done using a beverage containing one part of vodka (37.5%) and two parts of tonic. Depending on the participants’ body weight, which ranged from 57 to 99 kg, the served amount of vodka ranged from 85.4 to 149.3 ml (*M* = 117.61 ml, *SD* = 15.77 ml). This corresponds to two to five standard drinks (one standard drink is defined as 14 g of pure alcohol, National Institute on Alcohol Abuse and Alcoholism [NIAAA] 2005). The beverages were divided equally over two glasses.

Participants in the placebo condition (*n* = 26; weight range 60–128 kg) received two glasses of tonic containing no alcohol, with 4 ml of vodka sprayed on top to induce an alcohol odor.

We administered the alcohol in a double-blind manner. All participants were told that they would be given a moderate dose of alcohol. While research assistants were aware of the possibility of two conditions, one research assistant remained in the room with the participant during questionnaire and task completion, while another research assistant mixed the beverages and administered them to the participant (e.g., Weafer and Fillmore 2008; Craig et al. 2009; Kamboj et al. 2013). Participants finished each of the two glasses within 4 min, with the two glasses served 2 min apart (Weafer and Fillmore 2008).

### Procedure

Upon arrival in the lab, we provided perceivers with a detailed information sheet and offered to answer any participant question concerning the study. Individuals who verbally agreed to participate subsequently were asked to provide written informed consent.

A breathalyzer (Dräger Alcotest 7510) was administered at baseline to ensure that participants were sober. Body weight was recorded for calculation of the amount of alcohol or placebo. Upon consumption of both drinks, breath alcohol concentration (BAC) was recorded in 10-min intervals until the end of the experiment. To measure acute effects of alcohol, participants started the EA task immediately after beverage consumption, thus, on the ascending limb of the blood alcohol concentration curve. Following the EA task, participants were asked to complete several more questionnaires not relevant for the present report.

Upon completion of the questionnaires, participants answered debriefing questions about the perceived difficulty of the tasks. Subsequently, we administered a final breathalyzer test. Before leaving the lab, participants in both conditions estimated the amount of alcohol they had received. We provided participants in the alcohol condition with water and snacks while remaining in the lab until their BAC declined below 0.02 g%.

## Results

### Baseline and drinking data

Table 1 shows that the alcohol group had marginally higher r-AUDIT scores than the placebo group, but the two groups were otherwise very similar at baseline. Higher r-AUDIT scores were correlated with a higher average number of daily drinks, *r* = 0.75, *p* < 0.0001, and with a higher peak number of daily drinks, *r* = 0.67, *p* < 0.0001.

**Table 1:** Baseline and drinking data for the alcohol and placebo groups

	Placebo (*n* = 26)	Alcohol (*n* = 28)	*t* value	*p* value
Baseline data				
Age (years)	24.54 (5.44)	24.64 (7.32)	− 0.06	0.95
Weight (kg)	82.26 (18.10)	78.68 (10.52)	0.88	0.38
SIAS total score	16.31 (9.78)	15.43 (7.37)	0.37	0.71
Revised AUDIT score	9.61 (2.98)	11.61 (4.43)	− 1.92	0.06
DMQ-R scores				
- Sociability subscale	15.46 (4.00)	16.64 (4.61)	− 1.00	0.32
- Enhancement subscale	13.65 (4.54)	14.46 (4.64)	− 0.65	0.52
- Coping subscale	7.81 (2.10)	7.29 (2.81)	0.77	0.45
- Conformity subscale	6.50 (2.01)	6.18 (2.04)	0.58	0.56
Average number of daily drinks	4.13 (1.21)	4.36 (1.42)	− 0.62	0.54
Peak number of daily drinks	8.08 (4.12)	8.39 (3.60)	− 0.30	0.76
Drinking data				
Urge to drink				
- Baseline	4.88 (2.47)	4.89 (2.60)	− 0.01	0.99
- Right before drinking	4.92 (2.20)	4.82 (2.61)	0.15	0.87
- Right after drinking	4.92 (2.21)	4.43 (2.66)	0.74	0.46
- Right after EAT	5.38 (2.28)	5.57 (2.12)	− 0.31	0.75

*AUDIT* Alcohol Use Disorders Identification Test, *DMQ*-*R* Revised Drinking Motives Questionnaire, *EAT* Empathic Accuracy Task, *SIAS* Social Interaction Anxiety Scale

In the alcohol group, the mean alcohol dose was 117.60 ml (*SD* 15.77) and the mean peak breath alcohol concentration (BAC) was 0.25 mg/L (*SD* 0.06). This was significantly different from the placebo group, for which the mean peak BAC was 0 mg/L, *t*(27) = − 22, *p* < 0.0001. The median time to peak BAC in the Alcohol group was 20 min (*M* = 21, *SD* = 12). There were no differences in urge to drink at any time point between the alcohol and placebo groups (Table 1).

### Effect of drinking on empathic accuracy

The mean EA score (*r*) across all 864 participant/video clip combinations was 0.63 (range − 0.99 to + 1.00). In separate multilevel models, valence of clip was a significant predictor of EA, *F*(1,53) = 17.86, *p* < 0.0001, *d* = 1.16, and target sex was not, *F*(1,53) = 0.29, *p* > 0.59, *d* = 0.15. Participants obtained higher EA scores when watching positive clips (mean *r* = 0.67) compared to negative clips (mean *r* = 0.59). Participants obtained similar EA scores when watching clips of female targets (mean *r* = 0.62) compared to male targets (mean *r* = 0.63).

There was no significant main effect for condition, *F*(1,52) = 0.82, *p* > 0.36, *d* = 0.25. Controlling for valence did not change this. Further, when we analyzed the data for negative and positive film clips separately, the effect for condition was not significant for the negative clips, *F*(1,52) = 0.02, *p* > 0.89, *d* = 0.04, or for the positive clips, *F*(1,52) = 1.53, *p* > 0.22, *d* = 0.34. Furthermore, when we repeated the analyses with target sex added as a moderator, the results did not change.

### Hazardous drinking as a moderator

The mean r-AUDIT score in the sample was 10.65 (*SD* = 3.90). In a model including condition (alcohol, placebo), r-AUDIT scores (continuous factor, grand-mean centered), and the condition by r-AUDIT interaction, the effect for the interaction was significant, *F*(1,50) = 5.19, *p* < 0.03. Post-hoc tests indicated a difference in empathic accuracy between alcohol and placebo among participants with lower r-AUDIT scores, *t*(50) = − 2.44, *p* < 0.02, *d =* 0.69, and no significant difference between the two conditions among participants with higher r-AUDIT scores, *t*(50) = 1.02, *p* > 0.31, *d* = 0.29. In a second model, we controlled for valence and target sex; the result did not change.

**Fig. 1:**
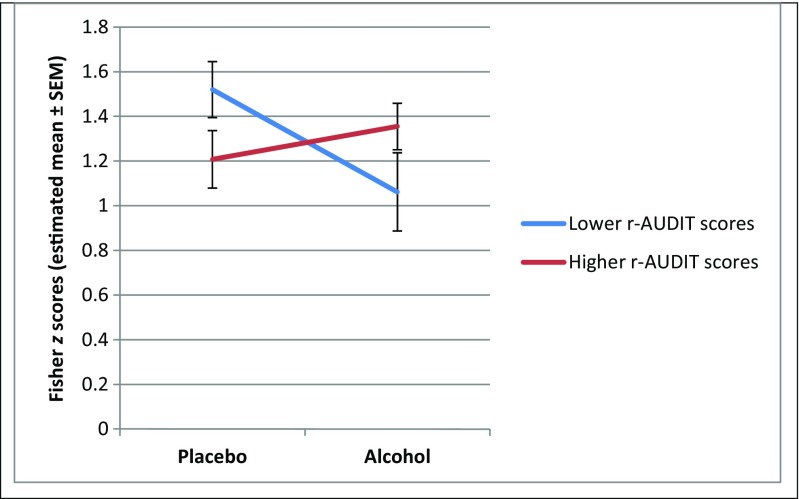
Empathic accuracy, expressed as Fisher *z* scores, for positive film clips among participants with lower (1 SD below mean) versus higher (1 SD above mean) r-AUDIT scores who received placebo or alcohol. Condition (placebo versus alcohol) was a between-subject factor

In a third and fourth model, we analyzed the data for the positive and negative film clips separately. The interaction was significant for positive film clips, *F*(1,50) = 4.55, *p* < 0.04, but not for negative film clips, *F*(1,50) = 1.19, *p* > 0.28. Post-hoc tests showed that the effect for condition was significant among participants with lower r-AUDIT scores, *t*(50) = −2.55, *p* < 0.02, *d =* 0.72, and not among participants with higher r-AUDIT scores, *t*(50) = 0.72, *p* > 0.47, *d* = 0.20. To illustrate this, Fig. 1 shows the negative slope for the effect for condition on EA for positive film clips in the group with lower r-AUDIT scores, and no negative slope in the group with higher r-AUDIT scores.

Overall, the results suggest that alcohol may selectively reduce empathic accuracy for positive events in individuals who do not drink hazardously according to the r-AUDIT. In individuals who drink hazardously, alcohol does not appear to alter empathic accuracy. At the suggestion of an anonymous reviewer, we added participants’ average or peak number of daily drinks as a covariate to all four models. The results did not change. Moreover, when we repeated the analyses using the average number of daily drinks in the last month as a moderator instead of r-AUDIT scores, the effect of condition was never significant. This suggests that the effects of alcohol on empathic accuracy are not moderated by excessive drinking per se (i.e., high drinking frequency and intensity) but rather by one’s risk for dependence and harmful use.

## Discussion

We examined the acute effects of a moderate dose of alcohol (0.56 g/kg) on empathic accuracy (EA). Overall, we did not find significant differences in EA between the alcohol and placebo conditions. This was unexpected as previous FER research suggests alcohol doses between 0.25–0.3 g/kg improve recognition of happy faces (Kano et al. 2003; Dolder et al. 2017), while higher doses cause impairments of facial emotion recognition (Kano et al. 2003). These alcohol-induced effects may not have been apparent in the current study due to between-study task differences. While FER tasks use static visual stimuli, our EA task included video clips that provided both visual and auditory information for more naturalistic inferences about how someone is feeling (see also Hogenelst et al. 2015a).

Nonetheless, it appears that alcohol affected EA in a subset of the participants in our study. We found a significant difference in EA between the alcohol and placebo conditions among participants who did not drink hazardously. This presumed effect of alcohol on EA was specific to positive target videos and not seen in the more hazardous drinkers, a finding that seems to be in line with the Low Level of Response Model (LLRM; Schuckit and Smith 2000; Morean and Corbin 2010). If those who do not drink hazardously respond to alcohol consumption by developing a positivity bias, this would explain their reduced accuracy for positive videos on the EA task. The idea that moderate doses of alcohol induce a positivity bias, or act as a social lubricant, has previously been reported (e.g., Monahan and Lannutti 2000; Kano et al. 2003; Dolder et al. 2017). If those who drink hazardously are less sensitive to this positivity bias at moderate doses of alcohol, then according to the LLRM, this might be due to increased tolerance to alcohol’s subjective effects. This interpretation of our finding, that EA for positive video clips was reduced after alcohol consumption in participants who do not drink hazardously, needs to be further investigated to draw definite conclusions.

An additional finding was that EA was higher for positive than for negative target videos. This finding is in line with previous research from FER tasks indicating that people are generally better at recognizing positive than negative emotions in others (Leppänen and Hietanen 2003). One potential reason is that positive facial expressions may be characterized by more homogeneous and less complex features than negative facial expressions (Leppänen and Hietanen 2003). The effect of increased accuracy for judging positive emotions has also been established using naturalistic videos (e.g., Zaki et al. 2008; Hogenelst et al. 2015b).

### Limitations and future directions

The current study has some limitations. Firstly, the sample consisted of Dutch male social drinkers, thereby, limiting our findings’ generalizability to women and individuals with alcohol use disorders. Previous research indicates gender differences in EA performance (Ickes et al. 2000) and in response to alcohol consumption (Mumenthaler et al. 1999), as well as reduced cognitive empathy in alcohol-dependent subjects (Castellano et al. 2015; Bora and Zorlu 2016; Erol et al. 2017). Nevertheless, further factors related to alcohol dependence remain to be investigated. For instance, family history of alcohol dependence has been shown to be a risk factor for the development of alcohol use disorders (Cservenka 2016).

Further, future studies may benefit from employing a control condition. Expectancy effects of alcohol consumption in the placebo condition may provide an explanation for not finding EA differences in the two conditions in the present study. Urge to drink did not differ between the two groups, which indicates the role of an expectancy effect. Expectancy effects could be tested by utilizing a control condition in which participants are knowingly sober (Testa et al. 2006). Nonetheless, we used a between-group design, rather than within-group design in order to maintain participant blindness.

In addition, we were unable to make use of items 9 and 10 of the AUDIT due to scale imputation errors. While the current findings of AUDIT scores as a moderator of the effect of alcohol on EA thus need replication, we found internal consistency of the revised AUDIT adequate. Scores on the revised AUDIT also correlated with drinking frequency and intensity, providing further support for reliability and validity of the current iteration of the measure to assess hazardous drinking.

## Conclusion

The current results suggest that alcohol consumption acutely reduces empathic accuracy for positive emotions in men who do not drink hazardously, while empathic accuracy for negative emotions appears unaffected, as are men who drink hazardously. Our findings concerning those who do not drink hazardously could either be associated with alcohol-induced impairments of EA for positive emotions, or a type of enhancement of social interaction resulting from an alcohol-induced positivity bias. The first explanation would suggest negative effects of a 0.56 g/kg alcohol dose toward which those who drink hazardously may have become tolerant. The second explanation on the other hand would indicate seemingly positive effects of alcohol, supporting its function as a social lubricant. Again, those who drink hazardously may have required a higher dose of alcohol to present this potential positivity bias as a result of tolerance. Thus, the underlying mechanism by which alcohol exerts its effects on social cognition remains to be further examined.

## Electronic supplementary material

Figure S1 (DOCX 220 kb)
