# Supplementary material for: A moderate dose of alcohol selectively reduces empathic accuracy
Source: Psychopharmacology (Berl). 2018 Feb 28;235(5):1479–86. doi: 10.1007/s00213-018-4859-y (PMC5920003; doi:10.1007/s00213-018-4859-y)

**Supplementary material**

**Figure S1. Flow Chart of Participant Inclusion/Exclusion and Group Allocation**


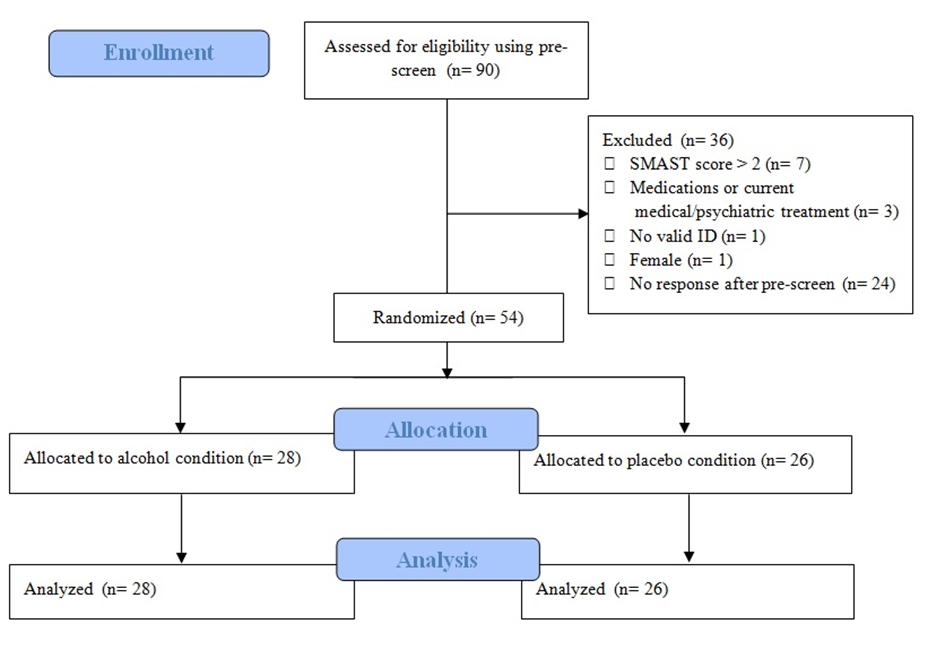

Supplement: Supplementary file 1 — (DOCX 220 kb) [file 213_2018_4859_MOESM1_ESM.docx]
